# Nuclear bodies reorganize during myogenesis in vitro and are differentially disrupted by expression of FSHD-associated DUX4

**DOI:** 10.1186/s13395-016-0113-7

**Published:** 2016-12-01

**Authors:** Sachiko Homma, Mary Lou Beermann, Bryant Yu, Frederick M. Boyce, Jeffrey Boone Miller

**Affiliations:** 1 Department of Neurology, Boston University School of Medicine, Boston, MA 02118 USA; 2 Department of Neurology, Massachusetts General Hospital, Boston, MA 02114 USA; 3 Neuromuscular Biology & Disease Group, Department of Neurology, Boston University School of Medicine, 700 Albany Street, Boston, MA 02118 USA

**Keywords:** DUX4, FUS, Facioscapulohumeral muscular dystrophy, Myotube, Nucleoli, PML bodies, SC35 speckles, TDP-43

## Abstract

**Background:**

Nuclear bodies, such as nucleoli, PML bodies, and SC35 speckles, are dynamic sub-nuclear structures that regulate multiple genetic and epigenetic processes. Additional regulation is provided by RNA/DNA handling proteins, notably TDP-43 and FUS, which have been linked to ALS pathology. Previous work showed that mouse cell line myotubes have fewer but larger nucleoli than myoblasts, and we had found that nuclear aggregation of TDP-43 in human myotubes was induced by expression of DUX4-FL, a transcription factor that is aberrantly expressed and causes pathology in facioscapulohumeral dystrophy (FSHD). However, questions remained about nuclear bodies in human myogenesis and in muscle disease.

**Methods:**

We examined nucleoli, PML bodies, SC35 speckles, TDP-43, and FUS in myoblasts and myotubes derived from healthy donors and from patients with FSHD, laminin-alpha-2-deficiency (MDC1A), and alpha-sarcoglycan-deficiency (LGMD2D). We further examined how these nuclear bodies and proteins were affected by DUX4-FL expression.

**Results:**

We found that nucleoli, PML bodies, and SC35 speckles reorganized during differentiation in vitro, with all three becoming less abundant in myotube vs. myoblast nuclei. In addition, though PML bodies did not change in size, both nucleoli and SC35 speckles were larger in myotube than myoblast nuclei. Similar patterns of nuclear body reorganization occurred in healthy control, MDC1A, and LGMD2D cultures, as well as in the large fraction of nuclei that did not show DUX4-FL expression in FSHD cultures. In contrast, nuclei that expressed endogenous or exogenous DUX4-FL, though retaining normal nucleoli, showed disrupted morphology of some PML bodies and most SC35 speckles and also co-aggregation of FUS with TDP-43.

**Conclusions:**

Nucleoli, PML bodies, and SC35 speckles reorganize during human myotube formation in vitro. These nuclear body reorganizations are likely needed to carry out the distinct gene transcription and splicing patterns that are induced upon myotube formation. DUX4-FL-induced disruption of some PML bodies and most SC35 speckles, along with co-aggregation of TDP-43 and FUS, could contribute to pathogenesis in FSHD, perhaps by locally interfering with genetic and epigenetic regulation of gene expression in the small subset of nuclei that express high levels of DUX4-FL at any one time.

## Background

During the formation of skeletal muscle, myoblasts stop proliferating and fuse with each other to form multinucleate myofibers. A large number of genes undergo changes in expression upon the myoblast to myofiber transition, and myofiber gene expression is often disrupted in muscle diseases. Many of the molecular mechanisms that underlie genetic and epigenetic regulation of skeletal muscle gene expression in normal development and in muscle disease are now understood in considerable detail [1–7], but questions still remain about gene regulation in both myogenesis and muscle diseases.

In this study, we show that multiple sub-nuclear structures (*i.e.*, nuclear bodies) reorganize during myotube formation in primary cultures of human myogenic cells. In addition, we further examine how nuclear bodies and additional nuclear proteins are affected by disease, using cultures of myogenic cells obtained from patients with muscle diseases. In particular, we examine myogenic cells obtained from donors with (i) congenital muscular dystrophy type 1A (MDC1A) due to laminin-alpha-2-deficiency, (ii) limb-girdle muscular dystrophy type 2D (LGMD2D) due to alpha-sarcoglycan-deficiency, and (iii) facioscapulohumeral muscular dystrophy (FSHD) type 1. FSHD type 1 is caused by genetic and epigenetic changes that promote aberrant expression of a full-length isoform of DUX4 (DUX4-FL), which is a highly cytotoxic transcription factor with a double homeodomain region [4, 8, 9]. A shorter isoform, DUX4-S, that lacks the C-terminal transactivation domain but retains the two homeodomains, is much less cytotoxic [10–12].

Nuclear bodies, such as the nucleoli, PML bodies, and SC35 speckles studied in this work, are dynamic sub-nuclear organelles that carry out different genetic and epigenetic processes of gene regulation [13, 14]. Nucleoli are sites of rDNA gene transcription, pre-rRNA processing, and initial pre-ribosome assembly; a previous study of mouse C_2_C_12_ cells showed that nucleoli were fewer in number but larger in size in myotube nuclei compared to myoblast nuclei [15]. PML bodies function in DNA repair, transcription, and protein stability, including in stress responses [13, 14], but little was known of PML bodies in myogenesis. Nuclear speckles that contain the SC35 protein include pre-mRNA splicing factors, and transcription sites for specific genes localize near SC35 speckles in myonuclei [16]. Additional gene regulation is provided by RNA/DNA handling proteins, notably TDP-43 and FUS, mutations of which have been linked to pathogenesis in some cases of amyotrophic lateral sclerosis (ALS) [17].

In a previous study, we found that expression of DUX4-FL, but not DUX4-S, induced nuclear, but not cytoplasmic, aggregates of TDP-43 [18]. DUX4-FL itself also forms aggregates in a subset of the nuclei in which it is expressed [18, 19], though DUX4-FL and TDP-43 do not appear to form co-aggregates [18]. To determine if DUX4-FL or TDP-43 co-aggregated with particular nuclear bodies or proteins, we have now carried out further studies on nucleoli, PML bodies, and SC35 speckles plus FUS. During these studies, we found that each of these three nuclear bodies reorganizes during myotube formation and that DUX4-FL expression can differentially disrupt nuclear body morphology and also lead to co-aggregation of FUS with TDP-43.

## Methods

### Cells and culture

All human cells used in this were obtained either from the Muscle Tissue Culture Collection (MTCC) at the University of Munich or from the Wellstone FSHD Cell Biobank which was at the Boston Biomedical Research Institute and is now located at the University of Massachusetts School of Medicine. The cells were anonymized prior to receipt with no personal identifying information available to us. The cells had been produced prior to our study from muscle biopsies collected under protocols approved by the appropriate institution that included informed donor consent and approval to publish results in accordance with standards of the Helsinki Declaration. Because our studies were of human cells that were obtained from a cell bank and for which personal identification data were not obtainable by us, the studies were classified as exempt from Human Studies review by the Boston University Institutional Review Board in accordance with the US Department of Heath and Human Services policy.

The human primary myogenic cells were grown on gelatin-coated dishes in high-serum medium for proliferation and were switched when near confluence to low-serum medium for differentiation as described [20, 21]. Myogenic cells from healthy control donors (07Udel, 09Ubic, 15Vbic, and 17Ubic) and from donors with FSHD type 1 (07Adel, 09Abic, 12Adel, 16Abic, 17Abic and 17Adel, 22Abic) or MDC1A (38/03, 50/04, 96/04) were as described previously [20–23]. Myogenic cells from LGMD2D donors were designated by the MTCC as 161/06 (3-year-old male donor with C100T Arg34Cys + C229T Arg77Cys mutations in *SCGA* encoding alpha-sarcoglycan) and 465/03 (5-year-old male donor with homozygous C229T Arg77Cys mutations in *SCGA*). When directly compared, we found no differences in the patterns of nuclear body reorganizations or FUS properties between cultures derived from different donors with the same disease or between cultures of healthy control cells that were derived from different donors.

To confirm authenticity, the primary cells were assessed for genetic mutation; SNP pattern [21]; expression of endogenous DUX4-FL in FSHD myotubes [21]; lack of laminin-a2 expression in MDC1A myotubes; and/or lack of expression or aberrant localization of a-sarcoglycan in LGMD2D myotubes [22, 23]. Cultures were also regularly assessed for myoblast proliferation rate, proportion of desmin-positive cells, and extent of myotube formation. All cultures were used at <45 total population doublings, which was well prior to the slowing of proliferation rate that occurred at ~55–60 population doublings under our culture conditions.

### Immunostaining

As in our previous studies [18], the cultures of differentiated cells were washed twice with PBS and then fixed with 2% paraformaldehyde (PFA) or ice-cold 100% methanol for 10 min as found to be appropriate for the primary antibody in preliminary validation experiments. Fixed cultures were washed three times with PBS. PFA-fixed cultures were additionally permeabilized with 0.5% Triton X-100 for 10 min at room temperature. All fixed cultures were blocked for 60 min at room temperature in 4% horse serum, 4% goat serum (Thermo Fisher), and 4% bovine serum albumin (EMD Millipore, Billerica, MA) in PBS plus 0.1% Triton X-100. Fixed and blocked cultures were incubated overnight at 4 °C with primary antibody diluted in blocking solution as noted below. The following day, the cells were rinsed three times with PBS and incubated for 1 h with the appropriate secondary antibody diluted 1:500 in blocking solution. For double immunostaining, the cultures were subsequently incubated as above with the second primary antibody, washed as above, and incubated with the second secondary antibody.

### Antibodies

DUX4-FL was detected with rabbit anti-DUX4-FL mAb E55 [10] used at 1:200 dilution (cat. ab124699, Abcam, Cambridge, MA). Myosin heavy chain (MyHC) isoforms were detected with mouse mAbs F59 [24] or MF20 [25] (Developmental Studies Hybridoma Bank, Iowa City, IA) used at 1:10 dilution of hybridoma supernatant or with rabbit anti-MYH3 pAb (cat. HPA021808, Lot A75757; Sigma-Aldrich, St. Louis MO) used at 1:500. Nucleolin was detected with a mouse mAb (cat. ab13541; lot GR217162-5 Abcam) used at 1:400. PML was detected with a mouse mAb (cat. sc-966, Santa Cruz Biotech, Dallas TX) used at 1:200. SC35 was detected with a mouse mAb (cat. ab11826, lot GR272322-1; Abcam) used at 1:1000. FUS was detected with a rabbit pAb (cat. 11570-1-AP, lot 00024677; ProteinTech, Rosemont Illinois) used at 1:200. TDP-43 was detected with either rabbit anti-TARDBP pAb (cat. 10782-2-AP; Proteintech) or mouse anti-TDP-43 mAb (cat. 60019-2; Proteintech) used at 1:200 dilution. Rabbit anti-PITX1 (Dixit et al.) was a gift of Dr. Yi-Wen Chen and was used at 1:500. V5 epitope tag was detected using either mouse anti-V5 mAb (cat. R960-25, Thermo Fisher) used at 1:500 or a rabbit pAb (cat. AB3792, EMD Millipore) used at 1:300. Each of the primary antibodies we used was validated based on one or more methods, including prior use in multiple published studies with the same mAb or lot of polyclonal antiserum, manufacturer’s validation assays including knockouts, generation of expected immunofluorescence staining patterns, detection of appropriate band size on immunoblots without detection of non-specific bands, and detection of recombinant protein when expressed in cells that normally do not express the protein. Primary antibody binding was visualized with appropriate species-specific secondary antibodies (Thermo Fisher) conjugated to either Alexa Fluor 488 or Alexa Fluor 594 and used at 1:500. Nuclei were stained with bisbenzimide.

### Microscopy

Images of immunostained cultures were acquired using a Nikon E800 microscope with a Spot camera and software version 5.1 (Diagnostic Instruments Inc., Sterling Heights, Michigan). Numbers of nuclear bodies were quantified either manually or with the counting application of the software. Cross-sectional areas of nuclear bodies were determined using the area application of the software to measure areas of manually delineated outlines of the structures. For different measurements, we cross-validated outcomes by showing that comparable results were obtained from healthy and/or diseased cell cultures by two or three independent observers. In addition, we verified that sample sizes were sufficient by showing in healthy and/or diseased cell cultures that similar results were obtained from analyses of multiple, independently identified groups of specified sample sizes.

### BacMam vectors

BacMam vectors were derived from pCMV-*DUX4-fl-V5* and pCMV-*DUX4-s-V5* [12, 18] by EcoRI/XbaI restriction digest and cloning of the corresponding *DUX4* inserts into pENTR1A (Life Technologies) which was prepared by digestion with EcoRI and XbaI. The resulting entry clones were recombined into the BacMam destination vector pJiF2 using LR ClonaseII (Life Technologies) and recombinant baculovirus generated using the Tn7 transposition system [26]. The PITX1 BacMam vector was prepared by Gateway recombinase reaction of the PITX1 clone BC003685 (obtained from the MGH Center for Computational and Integrative Biology) with the BacMam vector pHTBV1.1. The resulting plasmid was inserted into the baculoviral genome also using the Tn7 transposition system [26] and transfected onto Sf9 cells to generate recombinant baculovirus. In the BacMam vectors, expression was driven by a human CMV-IE1 promoter. P2 or P3 viral supernatants were used in all experiments without further purification and expression was analyzed at 24–48 h after addition.

## Results

### Nucleoli reorganize during myogenesis and are not affected by DUX4-FL

**Fig. 1:**
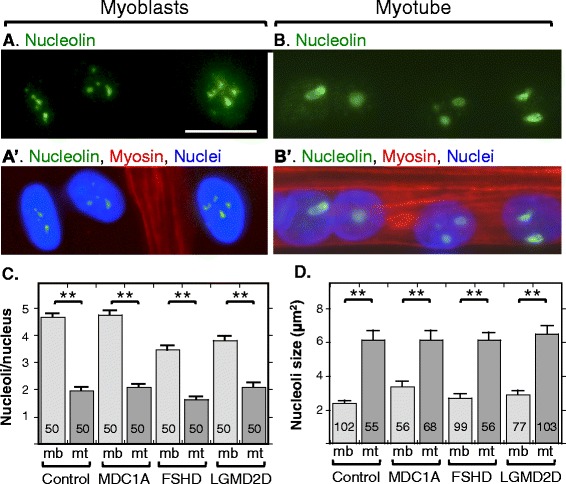
Myotubes had fewer, but larger, nucleoli than myoblasts. Immunostaining for nucleolin (*green*) was used to identify nucleoli and staining for myosin heavy chain (*red*) was used to distinguish nuclei in myotubes from those in myoblasts. **a**, **a’** Myoblast nuclei, three of which are shown, typically had four or five nucleoli. **b**, **b’** Myotube nuclei, of which four are shown from a single myotube, usually had one to three nucleoli that were typically larger than those in the myoblast nuclei. **c** Quantitation of nucleoli in myoblasts (*light gray bars*) and myotubes (*dark gray bars*) in cultures of healthy control, MDC1A, DUX4-negative FSHD, and LGMD2D myogenic cells. All cultures showed similar decreases in nucleolar number in myotube vs. myoblast nuclei. *Error bars* = s.e.m. **P < 0.01 by *t* test. Nucleoli were counted in *n* = 50 nuclei. **d** Quantitation of the cross-sectional areas of nucleoli in myoblasts (*light gray bars*) and myotubes (*dark gray bars*) in cultures of healthy control, MDC1A, DUX4-negative FSHD, and LGMD2D myogenic cells. All cultures showed similar increases in nucleolar size in myotube vs. myoblast nuclei. Scale bar in *A* = 20 μm. *Error bars* = s.e.m. **P < 0.01 by *t* test. Number of nucleoli measured as indicated on each data bar

In cultures of myogenic cells from human healthy controls, we identified nucleoli by immunofluorescence staining for nucleolin and found that myoblast nuclei typically contained about three to six small nucleoli, whereas myotube nuclei typically contained one to three nucleoli (Fig. 1a–c). For myoblasts, the mean ± s.e.m. number of nucleoli was 4.7 ± 0.13, and for myotubes, the number was 2.0 ± 0.12, which was significantly fewer than that in myoblasts (*P* < 0.01, *t* test, nucleoli were counted in *n* = 50 nuclei). This pattern is very similar to that found previously in cultures of the mouse C_2_C_12_ myogenic cell line where myoblast nuclei had an average of 5.3 nucleoli and myotube nuclei had an average of 1.7 [15]. In addition to decreasing in number, the nucleoli in myotubes formed from healthy control myoblasts were also larger on average than the nucleoli in myoblasts (Fig. 1d).

To determine if nucleolar reorganization was affected by disease, we examined nucleolar numbers and cross-sectional areas in cultures of myogenic cells from MDC1A, LGMD2D, and FSHD donors (Fig. 1c, d). In cultures of cells obtained from donors with each of these diseases, we found the same patterns of nucleolar reorganization as in healthy controls. That is, nucleoli in myotubes were fewer in number but larger in size than those in myoblasts. Thus, disease status did not affect nucleolar reorganization during myotube formation. We chose to examine these three diseases due to their distinct types of causative mutations and pathogenic mechanisms. MDC1A is due to mutation of an extracellular protein (laminin-a2), whereas LGMD2D is due to mutation of a sarcolemmal protein (a-sarcoglycan) and FSHD is due to genetic and epigenetic alterations that lead to aberrant expression of a nuclear transcription factor (DUX4-FL).

**Fig. 2:**
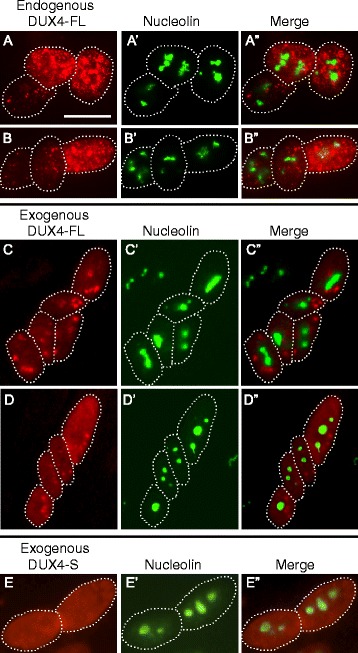
Nucleoli appeared to be unaffected by expression of DUX4-FL. **a**–**b”** Staining for endogenously expressed DUX4-FL (*red*) and nucleolin (*green*) in three nuclei within a single myotube. In these nuclei, DUX4-FL expression did not appear to affect nucleolar structure, and there was little or no overlap of punctate staining for DUX4-FL with nucleolin. In all panels, *dotted lines* indicate approximate borders of individual nuclei. **c**–**d”** Staining for endogenously expressed DUX4-FL (*red*) and nucleolin (*green*) in several nuclei within a single myotube. Exogenous DUX4-FL expression, whether predominantly punctate (**c**) or predominantly uniform (**d**) also did not appear to affect nucleolar structure, and there was also little or no overlap of exogenous DUX4-FL and nucleolin staining. **e** *–* **e”** When expressed in myotubes, the non-cytotoxic, short DUX-S isoform was uniformly distributed in nuclei and did not appear to affect nucleolar structure. Bar in *A* = 20 μm for rows **a**–**d** and 15 μm for row **e**

To determine if nucleolar structure might be affected in the small fraction (0.01–0.1%) of myotube nuclei that express DUX4-FL in FSHD cultures, we examined the effect of exogenous and endogenous DUX4-FL expression on nucleoli. To identify endogenous DUX4-FL expression, we examined differentiated cultures of myogenic cells obtained from FSHD patients with mAb E55 that is specific for the unique C-terminal region of DUX4-FL [10]. Consistent with previous studies [10, 21], DUX4-FL was expressed in only a small fraction of myotube nuclei and, within myotubes, there was typically a gradient of staining intensity among nuclei (Fig. 2a, b; but see Fig. 7a for a myotube with uniform intensity of DUX4-FL staining). As we noted in our previous study [18], endogenous DUX4-FL staining is often found in a punctate pattern in some nuclei (e.g., as in Fig. 2a, b) indicating aggregation but is more uniformly distributed in other nuclei. In different experiments, the percentage of nuclei with punctate DUX4-FL staining varied considerably for unknown reasons. In a typical study, however, about a quarter of the DUX4-FL-positive nuclei showed an obviously punctate DUX4-FL staining pattern.

For exogenous expression, we used a BacMam vector to express DUX4-FL in myoblasts and myotubes in cultures of healthy control myogenic cells as in our previous work [18]. Expression of exogenous DUX4-FL was identified with either the E55 mAb or with an anti-V5 mAb that recognizes a C-terminus epitope tag on the expressed protein. Consistent with our previous work, we found that a large fraction of nuclei showed staining for exogenous DUX4-FL at both 24 and 48 h after BacMam addition. Also, as for the endogenously expressed DUX4-FL, we found both punctate (Fig. 2c) and more uniform (Fig. 2d) staining patterns for exogenous DUX4-FL.

By double immunofluorescence, we did not find any obvious differences in nucleolar number or morphology in DUX4-positive vs. DUX4-negative nuclei (Fig. 2a–d), and this finding was the same for both endogenous and exogenous DUX4-FL and also for nuclei with punctate and uniform DUX4-FL staining patterns. Furthermore, in those nuclei with punctate DUX4-FL staining, the nucleolin and DUX4-FL stains did not overlap, indicating that DUX4-FL did not co-aggregate with nucleoli (Fig. 2a–c). BacMam-mediated expression of DUX4-S also did not alter nucleoli (Fig. 2e).

### PML bodies reorganize during myogenesis and a small fraction of PML bodies are disrupted by DUX4-FL

**Fig. 3:**
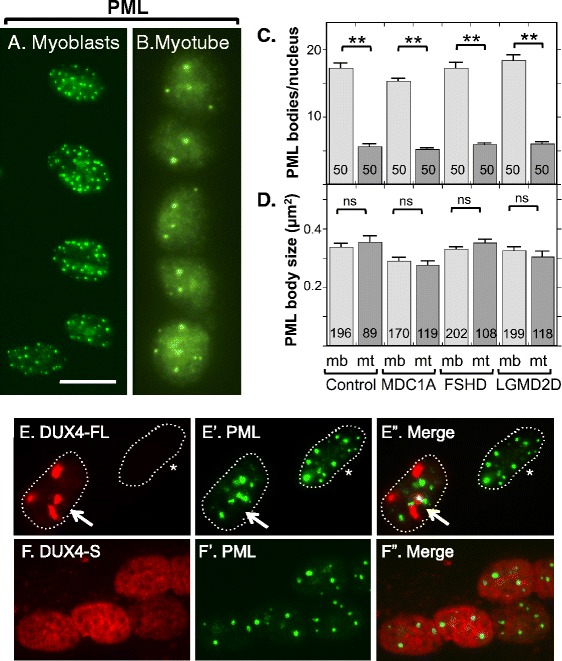
Myotubes had fewer PML bodies than myoblasts and the structure of most PML bodies was not affected by DUX4-FL expression. **a** Human myoblasts from a healthy donor typically had 10 to 20 or sometimes more PML-positive structures (*green*). **b** Nuclei in myotubes typically had four to eight PML bodies. **c** Quantitation of PML bodies in myoblasts (*light gray bars*) and myotubes (*dark gray bars*) in healthy control, MDC1A, DUX4-FL-negative, and LGMD2D myogenic cells. All cultures showed similar decreases in PML body number in myotube vs. myoblast nuclei. *Error bars* = s.e.m. **P < 0.01 by *t* test, with all PML bodies counted in *n* = 50 nuclei. **d** Quantitation of the cross-sectional areas of PML bodies in myoblasts (*light gray bars*) and myotubes (*dark gray bars*) in healthy control, MDC1A, DUX4-FL-negative FSHD, and LGMD2D myogenic cells. All cultures showed no significant changes in PML body size in myotube vs. myoblast nuclei. *Error bars* = s.e.m. *n.s.* not significant (*P* > 0.05) by *t* test. Number of PML bodies measured as indicated on each bar. **e–e”** In most, but not all (see Fig. 4), nuclei with punctate DUX4-FL fluorescence, PML bodies showed minor or no disruption, and there was little overlap between DUX4-FL and PML fluorescence. Nuclei in myotubes are shown. *Arrow* = DUX4-FL-positive nucleus, *asterisk* = DUX4-FL-negative nucleus. **f–f”** DUX4-S was typically uniformly distributed within the nuclei, and expression of DUX4-S did not affect PML body morphology. Nuclei in myotubes are shown. *Bar* in **a** = 20 μm for **a**, **b**, **f,** and 15 μm for **e**

We quantified nuclear PML bodies that were identified by immunofluorescence staining for the PML protein and found that myoblast nuclei typically contained about 15–20 small round PML bodies (Fig. 3a). Myotube nuclei, in contrast, usually had only four to eight PML bodies (Fig. 3b). Quantitation showed that cultures of myogenic cells from healthy controls, as well as from MDC1A, LGMD2D, and FSHD donors, all showed similar decreases in the number of PML bodies in myotube nuclei compared to myoblast nuclei (Fig. 3c). Unlike nucleoli, we did not find a difference in the average size of PML bodies between myoblasts and myotubes in healthy control, MDC1A, LGMD2D, or FSHD cultures (Fig. 3d). As an example, in a healthy control culture, PML bodies had an average cross-sectional area of 0.34 ± 0.16 μm^2^ in myoblasts (ave ± SD, *n* = 196) and 0.35 ± 0.19 μm^2^ in myotubes (ave ± SD, *n* = 81) (*P* = 0.47 by unpaired *t* test).

**Fig. 4:**
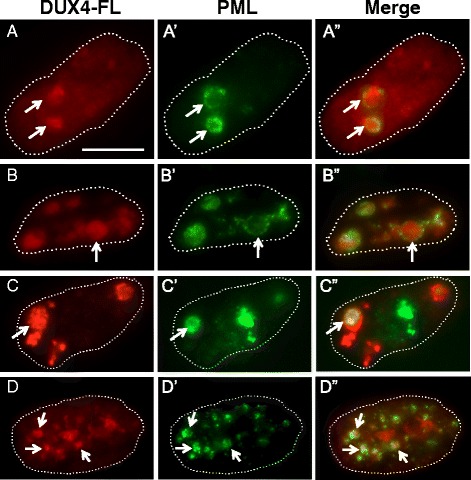
The structures of PML bodies in a small fraction of nuclei were disrupted by DUX4-FL expression. Each row shows one myotube nucleus immunostained as indicated for DUX4-FL or PML along with a merged image. *Dotted lines* show the approximate outlines of each nucleus. In rows **a–a”** and **b–b”**, the *arrows* point to regions where PML staining appears to envelop DUX4-FL aggregates; and in rows **c**–**c”** and **d**–**d”**, the *arrows* point to PML staining that appears to be intertwined with small DUX4-FL aggregates. *Bar* in **a** = 10 μm

Though disease status did not affect the overall pattern of PML body reorganization during myotube formation, DUX4-FL expression did appear to lead to disrupted organization of a small subset of PML bodies. In most nuclei that expressed endogenous or exogenous DUX4-FL, with either uniform and punctate staining, the PML bodies appeared to have normal morphology and there was little evidence of interaction between DUX4-FL and PML bodies (Fig. 3e) or between DUX4-S and PML bodies (Fig. 3f). However, in DUX4-FL-positive nuclei, there was a small fraction (~5–15% in different experiments) of nuclei that showed one or more PML bodies with disrupted organization, sometimes with close apposition to or intermingling with DUX4-FL aggregates (Fig. 4). For example, in some of these nuclei, PML staining appeared to be wrapped around DUX4-FL aggregates (Fig. 4a, b), and in others, PML appeared to be integrated within or wrapped around a group of small, closely spaced DUX4-FL aggregates (Fig. 4c, d).

### SC35 speckles reorganize during myogenesis and speckle patterns in most nuclei are disrupted by DUX4-FL

**Fig. 5:**
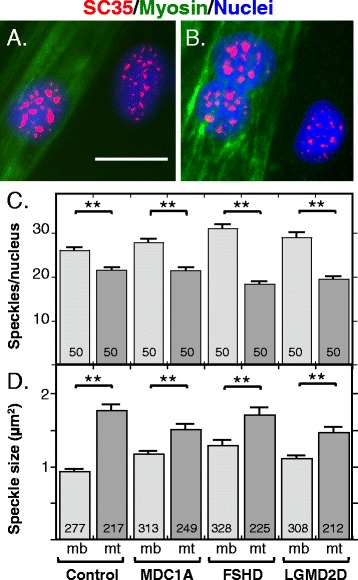
SC35-containing speckles in myotube nuclei were fewer in number but larger in size than those in myoblast nuclei. **a**, **b** Immunostaining for SC35 (*red*) was used to identify SC35 speckles and staining for myosin heavy chain (*green*) was used to identify myotubes and thus distinguish nuclei in myotubes from those in myoblasts. SC35 speckles in myotube nuclei typically appeared to be fewer in number and sometimes larger than those in myoblasts. **c** Quantitation of the number of SC35 speckles in myoblast nuclei (mb, *light gray bars*) and in myotube nuclei (mt, *dark gray bars*). As indicated, speckles were counted in healthy control, MDC1A, and LGMD2D myogenic cells, as well as in the DUX4-FL-negative nuclei of FSHD myogenic cells. In each type of cells, the average number of SC35 speckles was lower in myotube nuclei than in myoblast nuclei. *Error bars* = s.e.m. **P < 0.01 by *t* test. Speckles were counted in *n* = 50 nuclei. **d** Quantitation of the cross-sectional areas (μm^2^) of SC35 speckles in myoblasts (mb, *light gray bars*) and myotubes (mt, *dark gray bars*). As indicated, speckles were measured in healthy control, MDC1A, and LGMD2D myogenic cells, as well as in the DUX4-FL-negative nuclei of FSHD myogenic cells. In each type of cell, the average size of SC35 speckles was higher in myotube nuclei than in myoblast nuclei. *Error bars* = s.e.m. **P < 0.01 by *t* test. The number of speckles measured is indicated on each bar. *Bar* in **a** = 20 μm

We quantified SC35-containing speckles by immunofluorescence staining for SC35 and found that myoblast nuclei typically contained about 25–30 small speckles. Myotube nuclei, in contrast, usually had about 20 speckles, which was significantly less than the number in myoblasts (Fig. 5a–c). Myotube nuclei from MDC1A, LGMD2D, and FSHD patients all had significantly fewer SC35 speckles than myoblast nuclei and the decreases were of similar magnitudes (Fig. 5c). In addition, SC35 speckles in myotube nuclei had, on average, significantly larger cross-sectional areas than speckles in myoblast nuclei (Fig. 5d). Healthy control cultures, as well as MDC1A, LGMD2D, and FSHD cultures, all showed an increased speckle size in myotube vs. myoblast nuclei.

**Fig. 6:**
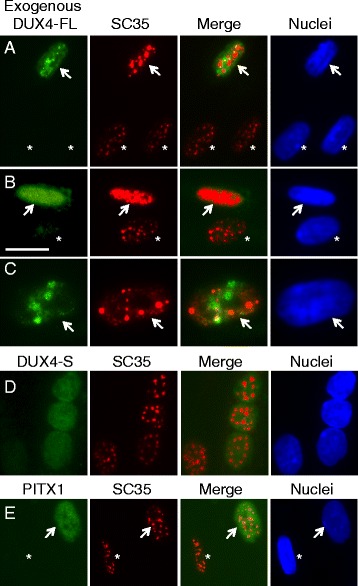
SC35 speckles in most nuclei were disrupted by exogenous DUX4-FL expression. **a–c** BacMam-mediated expression of DUX4-FL (*green*) in healthy control myotubes caused SC35 speckles (*red*) to show an altered morphology. *Arrows* indicate nuclei that expressed DUX4-FL, and *asterisks* indicate nearby nuclei that were DUX4-FL-negative. SC35 speckles in the DUX4-FL-positive nuclei typically showed larger aggregates and/or more intense staining. SC35 speckles were disrupted in both nuclei with punctate DUX4-FL staining (rows **a, c**) and in nuclei with uniform DUX4-FL staining (row **b**). In nuclei with punctate DUX4-FL (rows **a, c**), there was little or no overlap between DUX4-FL and SC35 staining. **d**, **e** In contrast to expression of DUX4-FL, exogenous, BacMam-mediated expression of DUX4-S (row **d**) or PITX1 (row **e**) did not markedly affect SC35 speckles in most nuclei. See Fig. 7e for quantitation of the extent to which exogenous DUX4-FL, DUX4-S, and PITX1 affects SC35 speckles using blind assays. *Bar* in **b** = 20 μm for **a**, **b**, **d**, and **e** and 12 μm for **c**

**Fig. 7:**
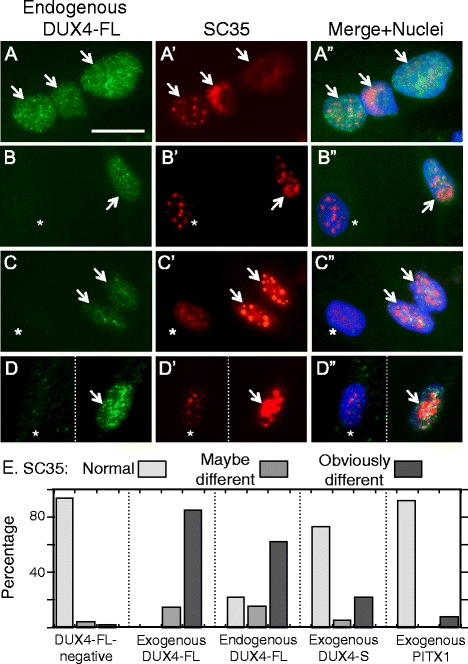
SC35 speckles were disrupted in many nuclei by expression of DUX4-FL from its endogenous promoter. **a–d”** Endogenous expression of DUX4-FL (*green*) in FSHD myotubes caused SC35 speckles (*red*) to show an altered morphology. *Arrows* indicate nuclei that expressed DUX4-FL, and *asterisks* indicate nearby nuclei that were DUX4-FL-negative. In panel **d**, the *dotted line* indicates where empty space was cropped from the image so that two nearby neighboring nuclei could be juxtaposed for presentation. The most common changes to SC35 speckles in the DUX4-FL-positive nuclei were the appearance of larger aggregates and/or more intense staining. Less common changes included loss of most speckles (e.g., *rightmost* nucleus in row **a**) and disorganized speckles (e.g., row **b**). SC35 speckles were disrupted in both nuclei with punctate DUX4-FL staining (rows **a**, **c**) and in nuclei with uniform DUX4-FL staining (row **b**). In nuclei with punctate DUX4-FL (rows **a** and **c**), there was little or no overlap between DUX4-FL and SC35 staining. Some nuclei also showed little effect of DUX4-FL (e.g., *leftmost* nucleus in row **a**). **e** Quantitation of SC35 speckle morphology. As described in the text, a blind test was used to classify speckle patterns into three groups: (i) similar to the majority of controls (normal, *light gray bars*), (ii) maybe different from controls (*medium gray bars*), or (iii) obviously different from controls (*dark gray bars*). Nuclei that expressed either endogenous or exogenous DUX4-FL had much higher frequencies of obviously different SC35 speckle patterns, compared to nuclei in healthy control cultures or to nuclei that expressed exogenous DUX4-S or PITX1. *Bar* in **a** = 20 μm

The morphology of SC35 speckles was altered in a majority of nuclei upon exogenous (Fig. 6a–c) or endogenous (Fig. 7a–d) expression of DUX4-FL. The pattern of SC35 speckles in DUX4-FL-negative nuclei showed little variation, with most nuclei containing 20–30 speckles of similar sizes and somewhat fuzzy outlines distributed throughout the nucleus (Fig. 5 and nuclei marked by asterisks in Figs. 6a–c and 7a–d). In contrast, SC35 speckles in exogenous or endogenous DUX4-FL-positive nuclei showed an altered range of morphologies and numbers. In particular, the most frequent change in DUX4-FL-positive nuclei was the appearance of one or more very large, irregularly shaped regions of intense SC35 staining (Figs. 6b, 7a middle nucleus, d), and the second most common change was the appearance of a reduced number of larger than usual, round speckles (Figs. 6a, c and 7c). Other abnormal patterns that were uncommon included a relatively uniform, low-intensity staining (Fig. 7a, rightmost nucleus) and an open, disorganized staining (Fig. 7b). In addition, as noted in the blind test, SC35 speckle staining did not appear to be significantly affected in some DUX4-FL-positive nuclei (Fig. 7a, leftmost nucleus)

To further assess this result, we carried out a blind test to confirm that the SC35 staining patterns in DUX4-FL-positive and DUX4-FL-negative nuclei were distinctive. For this test, observers were provided with unlabeled images of SC35 immunofluorescence and were asked to classify the SC35 staining pattern in each nucleus as “obviously different,” “maybe different,” or “the same as” the pattern seen in the healthy control cells. Each image contained many nuclei (range 12–1, ave = 19.8, total = 1109). After the observers had classified all nuclei, the SC35 images were compared to companion images of the same fields that had been immunostained for DUX4-FL, expressed either from the BacMam vector or from its endogenous promoter. The study was designed so that about half of the images had no DUX4-FL-positive nuclei, whereas the remaining images had one or more. (For this study, we used an amount of BacMam that generated expression in about 5% of the nuclei in these cultures of healthy control cells.) For BacMam-mediated expression of DUX4-FL, the observers classified 28 of the 33 (85%) DUX4-FL-positive nuclei in the blind image set as obviously different, and the remaining five DUX4-FL-positive nuclei were classified as maybe different, whereas only one or two nuclei would have been expected to be correctly identified by chance (*P* < 0.001 by Fisher’s exact test, observed vs. expected). For endogenous DUX4-FL expression, the observers classified 20 of the 32 (62%) DUX4-FL-positive nuclei as obviously different, five as maybe different (16%), and seven (22%) as normal (*P* < 0.001 by Fisher’s exact test, observed vs. expected). In contrast, only 25 of the 1010 (2%) DUX4-FL-negative nuclei were classified as obviously different and only 43 (4%) were classified as maybe different. These results, which are presented as a graph in Fig. 7e, implied that the SC35 speckles in DUX4-FL-expressing cells were sufficiently different from those in DUX4-FL-negative cells to be accurately identified in a majority (~60–80%) of the cases.

As further controls, we used a blind assay to examine SC35 speckles in nuclei that expressed either DUX4-S or PITX1 from a BacMam vector. DUX4-S was chosen because it is a non-cytotoxic protein with the same DNA-binding domain as DUX4-FL. PITX1 was chosen because it is a homeodomain-containing transcription factor that has been proposed to be regulated by DUX4-FL and to perhaps play a role in FSHD pathology [27], though this possibility is contested [28]. Observers classified nuclei as having obviously different SC35 staining in 11 out of 50 (22%) DUX4-S-expressing nuclei and 1 out of 16 (6%) PITX1-expressing nuclei (Fig. 7e). Thus, most nuclei that expressed exogenous DUX4-S or PITX1 had SC35 speckles of normal morphology and number (Fig. 6d, e). However, DUX4-S expression did appear to affect SC35 speckles in a subset of nuclei, though the effect of DUX4-S was less pronounced than that of DUX4-FL. Exogenous expression of PITX1 appeared to have no effect on SC35 speckles.

### DUX4-FL-induced co-aggregation of FUS with TDP-43

**Fig. 8:**
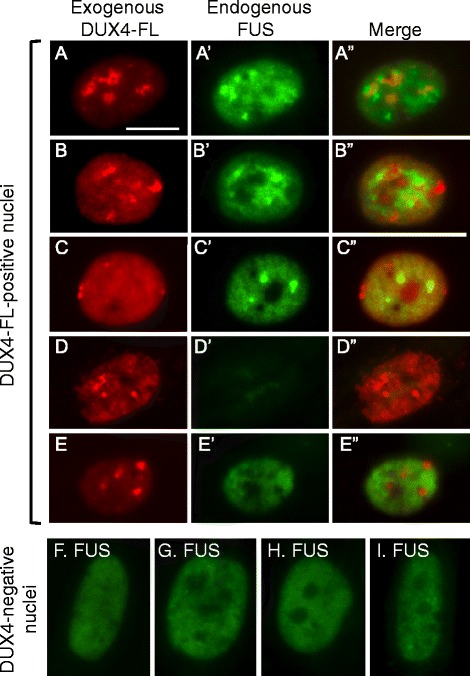
Exogenous DUX4-FL expression induced abnormalities in FUS expression. At 24 h after addition of BacMam vector, healthy control myotubes were examined for expression of exogenous DUX4-FL (*red*) and FUS (*green*). **a–c”** About 40–50% of the DUX4-FL-positive myotube nuclei showed punctate immunostaining for FUS (panels **a**–**c**). In addition, DUX4-FL itself showed punctate staining in some nuclei and merged images indicated that FUS and DUX4-FL puncta were not usually co-localized. **d–d”** About 10% of the DUX4-FL-positive myotube nuclei showed little or no staining for FUS (panel **d**). **e–e”** The remaining approximately half of the DUX4-FL-positive myotube nuclei showed a more uniform distribution of FUS staining in the nucleus (though excluded from nucleoli), even when DUX4-FL staining was itself punctate. **f**–**i** For comparison, myotube nuclei that did not express DUX4-FL typically showed the more uniform pattern of FUS staining (*green*) without large puncta, which was similar to the nucleus in panel **e**. Bar in **a** = 10 μm for **a**–**e**” and 8 μm for **f**–**i**

**Fig. 9:**
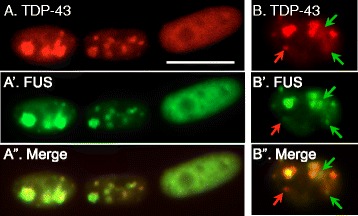
Exogenous DUX4-FL expression induced nuclear co-aggregates of FUS and TDP-43. **a-a”** Double immunostaining of TDP-43 (*red*) and FUS (*green*) in myotubes which also expressed exogenous DUX4-FL showed that TDP-43 and FUS staining were almost completely co-localized. **b–b”** In rare myotube nuclei, some TDP-43 puncta (*red arrows*) and FUS puncta (*green arrows*) did not co-localize with the other protein, even though most of the FUS and TDP-43 in the same nucleus were co-localized. *Bar* in **a** = 10 μm

Our previous study [18] showed that DUX4-FL expression induces nuclear aggregates of TDP-43. Because TDP-43 is found in co-aggregates with FUS in ALS [17], we sought to determine if FUS was also affected by DUX4-FL expression. We found that FUS nuclear aggregates were induced by expression of DUX4-FL, either exogenously from a BacMam vector (Fig. 8) or from its endogenous promoter (Fig. 9), and that FUS co-aggregated with TDP-43.

When DUX4-FL was exogenously expressed in healthy control cultures, ~40–50% of myotube nuclei showed an altered pattern of FUS immunostaining. The altered FUS staining in DUX4-FL-positive nuclei was characterized either by the presence of puncta or by a nearly complete loss of FUS staining (Fig. 8a–d) which contrasted with the uniform stain in DUX4-negative nuclei (Fig. 8f–i). In one experiment, for example, we examined 130 DUX4-FL-positive nuclei and found 40 (31%) with punctate nuclear staining (Fig. 8a–c); 14 (11%) with little or no FUS staining (Fig. 8d); and 76 (58%) with nearly uniform FUS staining (Fig. 8e). Punctate FUS staining was seen in some nuclei with uniform DUX4-FL staining (Fig. 8c); in those nuclei with a punctate pattern of DUX4-FL, the FUS and DUX4-FL signals showed little or no overlap (Fig. 8a, b). In contrast, FUS puncta overlapped almost completely with TDP-43 puncta in DUX4-FL-expressing nuclei (Fig. 9), suggesting that TDP-43 and FUS formed co-aggregates.

The frequency of FUS puncta or decreased FUS signal intensity was significantly higher in DUX4-FL-positive than DUX4-FL-negative nuclei. In myoblasts and myotubes of healthy controls, FUS immunostaining was usually excluded from nucleoli but was otherwise uniformly distributed throughout the nucleus (Fig. 8f–i). In one survey of 560 DUX4-FL-negative myotube nuclei in healthy control cultures, for example, we found only 17 nuclei (3%) with puncta and 5 nuclei (1%) with little or no staining, a result that was significantly different from that noted above for nuclei that expressed exogenous DUX4-FL (*P* < 0.001, chi-square). Myoblasts and myotubes in MDC1A and LGMD2D cultures showed FUS staining patterns that were similar to those of healthy controls; and, in FSHD cultures, the large majority of myotube nuclei that did not immunostain for DUX4-FL also had the same pattern of FUS staining as healthy controls (not shown).

**Fig. 10:**
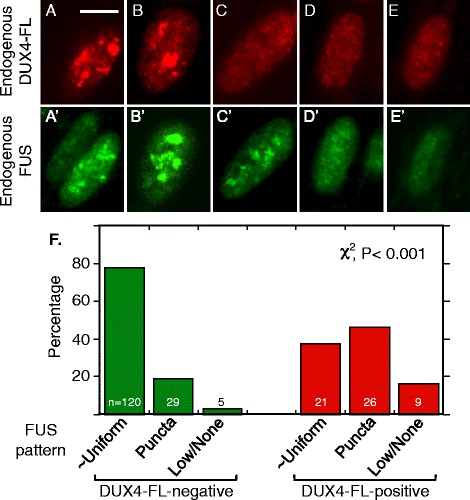
Induction of nuclear aggregates of FUS by endogenous expression of DUX4-FL in FSHD myotubes. **a–e”** Nuclei were double stained for endogenous DUX4-FL (*red*, *top row*) and endogenous FUS (*green*, *lower row*) in FSHD myotubes. The five nuclei illustrate a range of staining patterns ranging from mostly large, though non-overlapping, puncta for both proteins (e.g., **a**, **b**) to more uniform staining or very small puncta (e.g., **d**, **e**). **f** Quantitation of blind assays in which observers classified FUS staining patterns in DUX4-negative and DUX4-positive myotube nuclei as (i) mostly uniform (as illustrated in Fig. 7e–i), (ii) consisting largely of punctate staining (e.g., as in **a**, **b** of this figure), or (iii) showing a low intensity signal or no signal (e.g., as in Fig. 9d). Expression of DUX4-FL was associated with a significantly increased percentage of nuclei in which FUS showed a punctate staining indicative of aggregation or a loss of signal intensity. Bar in **a** = 10 μm

Aggregation of FUS was also induced in nuclei that expressed DUX4-FL from its endogenous promoter (Fig. 10). We found punctate staining for FUS both in nuclei where the staining for endogenous DUX4-FL was punctate (Fig. 10a, b) and in nuclei where DUX4-FL staining was more uniform (Fig. 10c). In those nuclei with punctate staining for endogenous DUX4-FL, there was little or no overlap between the FUS and DUX4-FL puncta (Fig. 10a, b). As for exogenous DUX4-FL, about half of the nuclei that expressed endogenous DUX4-FL continued to show a nearly uniform pattern of FUS nuclear staining (Fig. 10d, e). In those rare FSHD myotube nuclei that expressed endogenous DUX4-FL, a blind assay showed that the percentage of nuclei with punctate or low staining for FUS was significantly increased compared to the DUX4-FL-negative nuclei in the same culture (Fig. 9f).

## Discussion

In this study, we found that three different nuclear bodies—nucleoli, PML bodies, and SC35 speckles—undergo reorganization between the myoblast and myotube stages of human myogenesis in vitro. In each case, the number of bodies was decreased in myotube nuclei compared to myoblast nuclei. In addition, nucleoli and SC35 speckles were generally larger in myotubes than myoblasts. Reorganization of these nuclear bodies during normal development is consistent with a role for these structures in regulation of the gene expression changes that take place during myotube formation. In addition, we found that expression of DUX4-FL in human myogenic cells, either from its endogenous promoter or exogenously, disrupted the structure of a small fraction of PML bodies and a majority of SC35 speckles. DUX4-FL expression also induced FUS to co-aggregate with TDP-43 in a substantial fraction of nuclei. Because aberrant DUX4-FL expression, particularly in skeletal muscle, appears to be causative in FSHD, our results suggest that DUX4-FL-induced disruption of nuclear bodies and nuclear aggregation of FUS with TDP-43 may contribute to FSHD pathogenesis.

Our results showing nucleolar reorganization in human myogenesis are consistent with previous studies of non-human systems. For example, we found that the average number of nucleoli in human myogenic cells was ~4.7 in myoblasts and ~1.9 in myotubes, and a previous study of the mouse C_2_C_12_ cell line found nearly the same average number of nucleoli at ~5.3 in myoblasts and ~1.7 in myotubes [15]. Also, as in the human myotubes, the nucleoli in C_2_C_12_ myotubes were found to be larger than those in myoblasts [15]. In addition, rat soleus myofibers had an average of 1.6–1.7 nucleoli [29] and chicken myotubes in culture had an average of 1.2–1.9 nucleoli/myonucleus, with the lower number at longer durations of culture [30]. We additionally found that nucleolar reorganization during myogenesis was not affected in MDC1A, LGMD2D, or FSHD patient cells or by DUX4-FL expression. The number and size of nucleoli are thought to be related to cell size, stage of cell cycle, and biosynthetic requirements [31–33], but it remains to be determined whether these factors contribute to changes in nucleolar number and size during myogenesis.

As with nucleoli, we found that there were fewer PML bodies in myotube nuclei (usually 4–8) compared to myoblast nuclei (usually 15–20), and this reorganization was similar in MDC1A and LGMD2D cells, as well as in DUX4-negative FSHD myotubes. Unlike nucleoli, there was no average change in PML body size or morphology between myoblasts and myotubes. The PML protein and PML bodies have been shown to function in a number of processes relevant to myogenesis. For example, though skeletal muscle development is not markedly affected in *Pml−/−* mice, expression of muscle metabolic genes is altered, as is the regulation of cell growth and the retinoic acid pathway [34, 35]. It may be of particular relevance that PML is a regulator of p53-mediated cell death [36]. We show here that, in a small subset of myonuclei, PML appeared to interact with aggregates of DUX4-FL so that PML body structure was disrupted; and others have shown that DUX4-FL induces cell death through a p53-dependent pathway [37, 38]. Furthermore, DUX4-FL-mediated cell death is prevented by treatment of cells with arsenic trioxide, a drug that inhibits p53-mediated cell death by inhibiting the assembly of PML bodies [39].

Though the number of SC35 speckles decreased in myotube compared to myoblast nuclei, the ~20–25% reduction in speckle number was of smaller magnitude than the ~60–70% reductions in nucleoli and PML bodies. A figure in a previous study showed a similar change of SC35 speckles in mouse C_2_C12 myoblasts vs. myotubes, but the difference was not quantified or commented on [40]. The effect of DUX4-FL expression on SC35 speckles was much more extensive than the minor effect on PML bodies, as a majority of DUX4-FL-expressing nuclei showed altered SC35 staining. In nuclei with a punctate pattern of DUX4-FL staining, there was little or no overlap of SC35 and DUX4-FL, suggesting that these proteins did not co-aggregate.

SC35 (also known as SRSF2) is a member of the serine/arginine-rich (SR) family of pre-mRNA splicing factors. During the redistribution of gene loci that occurs within myotube nuclei upon differentiation [41, 42], muscle-specific genes become juxtaposed to the periphery of SC35 domains [16]. This juxtaposition suggests that SC35 may play a role in the alternative splicing switching that occur during myogenesis, as shown for beta-tropomyosin [43]. Splicing patterns are also markedly altered by DUX4-FL expression [39, 44]. Ventricle-specific knockout of SC35 in developing mouse cardiomyocytes leads to hypertrophy and impaired excitation-contraction coupling [45], though SC35 knockout in adult cardiomyocytes unexpectedly had no effect [46]. The role of SC35 in skeletal muscle has not been tested, though, in proliferating embryonic fibroblasts, SC35 knockout increased genomic instability and p53 activation [46]. Thus, it is possible that the DUX4-FL-induced alterations in SC35 speckles could have functional consequences in one or more of several possible pathways relevant to FSHD pathology, including p53-mediated cell death and disruption of alternative splicing patterns.

In addition to the DUX4-FL-induced changes in SC35 speckles, we found that DUX4-FL expression led to the formation of FUS aggregates in nuclei. In our previous study, we found that DUX4-FL induced similar nuclear aggregates of TDP-43 [18]. Here, we showed that the FUS and TDP-43 nuclear aggregates had almost completely overlapping staining patterns indicating likely co-aggregation as often seen in ALS tissue [47, 48]. DUX4-FL, in contrast, does not appear to co-aggregate with FUS (this work) or with TDP-43 [18]. FUS, like TDP-43, can bind to thousands of RNAs, as well as to single- and double-stranded DNAs; FUS regulates multiple stages of gene expression including transcription and splicing.

The extent to which aggregation of FUS and TDP-43 leads to gain or loss of function of either protein [48–51] and contributes to DUX4-FL-induced pathology remains to be determined. One approach would be to compare transcriptomes and splicing patterns in DUX4-FL-expressing myogenic cells with those in FUS and TDP-43 overexpression and knockdown cells. In our studies, we found that both exogenous and endogenous DUX4-FL induced only nuclear aggregates of the endogenously expressed FUS protein. We did not see the cytoplasmic FUS aggregates found in ALS. It will be informative to identify any additional proteins that may co-aggregate with FUS and TDP-43 in muscle nuclei [52, 53] and to determine if the FUS and TDP-43 in DUX4-FL-induced nuclear aggregates are post-translationally modified [54, 55]. Because endogenous DUX4-FL expression does not activate caspase-3 to a level detectable by immunofluorescence under our culture conditions [15], it appears that the nuclear aggregation of FUS did not require cell death activation or caspase-3-mediated cleavage of TDP-43 itself [56].

Our study adds new details to our understanding of nuclear reorganization during normal myogenesis and to the potentially pathological effects of aberrant DUX4-FL expression in FSHD. Nonetheless, our study has several limitations and many questions remain open. For example, it will be important to examine developing and regenerating muscles to determine if reorganization of nucleoli, PML bodies, and SC35 speckles occurs in vivo. Though we did not have access to appropriate human biopsies (e.g., embryonic or regenerating muscles) for such a study, several published studies have noted that the nuclei in embryonic and mature myofibers, including in humans, tend to have only one or two large nucleoli [57–60], which is consistent with our results in vitro. For example, Fig. 1 in Webb [57] shows a longitudinal section of human muscle at 12 weeks in utero in which myotube nuclei consistently show one or two nucleoli. Unfortunately, the myoblasts in that figure were overstained, so nucleoli are not discernible; we have not found other published images that show nucleoli in proliferating myoblasts in vivo at high resolution or with appropriate stains. We have also not found published images of PML bodies or SC35 speckles in skeletal muscles in vivo. Thus, in vivo analyses of nuclear bodies in skeletal muscle development and disease remain for further work.

It should also be informative to examine additional nuclear bodies, e.g., SMN gems, coilin bodies, to determine if these bodies also reorganize during myogenesis. Experiments in which the size or numbers of nuclear bodies are altered, perhaps by regulating expression of the component *Alu*RNAs [61] or proteins, could illuminate the functional consequences of the size and number changes. In addition, it will be informative to examine FSHD muscle biopsies for signs of PML body and SC35 speckle dysfunction or co-aggregation of FUS with TDP-43. If such signs of pathology are found, it would then be necessary to determine if the changes correspond to sites of concurrent DUX4-FL expression or are more widespread. To develop therapies for FSHD, several groups have developed a number of techniques to inhibit the function or expression of DUX4-FL [37, 62, 63]. Inhibition of DUX4-FL function or expression, if successful in patients, might also be expected to normalize structures of PML bodies and SC35 speckle structures and to prevent nuclear co-aggregation of FUS and TDP-43.

## Conclusions

Nucleoli, PML bodies, and SC35 speckles reorganize during human myotube formation in vitro. These nuclear body reorganizations are likely needed to carry out the distinct gene transcription and splicing patterns that are induced upon myotube formation. DUX4-FL-induced disruption of some PML bodies and most SC35 speckles, along with co-aggregation of TDP-43 and FUS, could contribute to pathogenesis in FSHD, perhaps by locally interfering with genetic and epigenetic regulation of gene expression in the small subset of nuclei that express high levels of DUX4-FL at any one time.
